# Anthocyanin Enhances Development, Hatching, and GLUT4 Expression in In Vitro-Cultured ICR Mouse Blastocysts

**DOI:** 10.3390/life16010132

**Published:** 2026-01-15

**Authors:** Imran Khan, Yun Seok Heo

**Affiliations:** 1 Animal Biotechnology Division, National Institute of Animal Science, Rural Development Administration, 1500 Kongjwipatjwi-ro, Wanju-gun 55365, Republic of Korea; imrangnu@gmail.com; 2 Department of Mechanical & System Design Engineering, Hongik University, 94 Wausan-ro, Mapo-gu, Seoul 04066, Republic of Korea

**Keywords:** anthocyanin, mouse blastocysts, embryo development, implantation-related processes, GLUT4, PI3K, cell proliferation

## Abstract

Anthocyanin is a flavonoid known for its strong antioxidant and anti-inflammatory activities in both in vitro and in vivo systems. This study investigated whether anthocyanin supplementation could improve the developmental competence, hatching rate, and the expression of development- and proliferation-related markers in ICR mouse blastocysts cultured in vitro. Mouse embryos were cultured in KSOM medium supplemented with 2, 4, or 8 μM anthocyanin. Among these, 4 μM was selected as the working concentration within the tested range. Morphological assessment was used to evaluate blastocyst development and hatching, while quantitative real-time polymerase chain reaction (qPCR) was performed to measure the expression of GLUT4 and PI3K. Anthocyanin supplementation significantly enhanced blastocyst quality, as reflected by higher developmental competence and increased hatching rates compared with the control group. In addition, anthocyanin-treated blastocysts displayed elevated mRNA expression of GLUT4 and PI3K, indicating a potential association with enhanced metabolic readiness and cellular proliferation. Overall, these findings indicate that anthocyanin supports embryo quality during preimplantation development in vitro, with potential relevance to implantation-related processes. Further research is needed to clarify the underlying mechanisms and explore the potential applications of anthocyanin in reproductive medicine.

## 1. Introduction

The efficiency of assisted reproductive technologies (ART) is strongly influenced by the optimization of in vitro culture conditions. Significant differences exist between in vitro and in vivo environments, particularly in oxygen tension [1]. High oxygen levels in vitro increase the production of reactive oxygen species (ROS), which can damage developing embryos by activating apoptosis-related signaling pathways such as P53 and P38 and by inducing DNA and protein damage [2]. Consequently, various antioxidants have been incorporated into culture media to mitigate oxidative stress and improve embryo development [3,4].

Ambient oxygen levels (20%) have also been shown to alter gene expression and protein profiles in mouse blastocysts and to disrupt carbohydrate and amino acid metabolism during early development [5,6]. Embryonic development and implantation are critical processes for establishing a successful pregnancy, and they are influenced by multiple factors, including diet, environment, and genetics [7,8]. Among molecular regulators, phosphoinositide 3-kinase (PI3K) and glucose transporter type 4 (GLUT4) play central roles in early embryo metabolism and implantation. PI3K regulates cell proliferation, survival, and metabolic activity, whereas GLUT4 mediates glucose uptake and serves as an important indicator of implantation competence [9,10].

Anthocyanins are naturally occurring pigments found in foods such as berries, grapes, and red cabbage. They possess strong antioxidant properties and various health-promoting effects, yet their influence on embryonic development remains poorly understood [11]. Recent evidence suggests that anthocyanins may interact with metabolic signaling pathways, including PI3K, and modulate the expression of genes such as GLUT4, potentially enhancing embryo development and implantation [12]. As interest grows in understanding how dietary bioactive compounds influence reproductive performance, anthocyanins have emerged as promising candidates for improving embryo quality in both animal breeding and reproductive medicine [13].

ICR (Institute of Cancer Research) mice are widely used in reproductive biology research due to their stable genetic background and well-characterized reproductive physiology [14]. They represent an appropriate model for evaluating the effects of anthocyanins on early embryonic development. Therefore, this study aimed to investigate how anthocyanin supplementation influences the developmental competence and hatching rate of ICR mouse blastocysts cultured in vitro, with particular emphasis on GLUT4 expression as a key implantation-related marker [15]. Additionally, we examined whether anthocyanins modulate PI3K signaling, which could further contribute to enhanced embryo development and implantation-related processes.

In this study, anthocyanins were introduced into KSOM medium for the first time to evaluate their effects on preimplantation embryo development. Three concentrations (2, 4, and 8 μM) were tested, and 4 μM was identified as the most effective, significantly improving blastocyst development, hatching, and implantation-related gene expression.

## 2. Materials and Methods

### 2.1. Experimental Design

The objective of this study was to determine the beneficial effects of anthocyanin on the in vitro development of ICR mouse blastocysts. To identify the optimal concentration, anthocyanin was supplemented into KSOM medium (KSOM; Merck, Darmstadt, Germany; Cat. No. MR-121-D) at 2, 4, and 8 μM. Based on developmental outcomes, 4 μM was determined to be the most effective concentration. Subsequent analyses were conducted by comparing the 4 μM anthocyanin group with the untreated control group to evaluate developmental competence and hatching rates.

### 2.2. Animals

ICR mice were purchased from Hana Corporation (Seoul, Republic of Korea). Male mice were 7–12 weeks old (30–40 g), and female mice were 5–7 weeks old (21–25 g). A total of 125 female ICR mice were used for embryo collection. Mice were maintained at the Laboratory Animal Research Center, Keimyung University, South Korea, in individually ventilated cages under controlled temperature (22 ± 2 °C), humidity (55 ± 5%), and a 12 h light/dark cycle, with ad libitum access to food and drink.

All animal procedures were examined and authorized by the Institutional Animal Care and Use Committee (IACUC) of Keimyung University (Approval No. KM-2022-12R1). Females were terminated post-mating using CO_2_ inhalation followed by cervical dislocation to ensure minimal pain. Pain management measures were not required, as only minimally invasive handling was performed prior to euthanasia.

### 2.3. Anthocyanin Preparation

Anthocyanin powder was dissolved in deionized water to prepare a 100 μM stock solution, which was subsequently diluted to the working concentrations of 2, 4, and 8 μM. For droplet culture, anthocyanin was diluted directly into KSOM medium. All media were sterilized using a 0.22 μm filter before use. According to the manufacturer’s specifications, the pure anthocyanin used in this investigation was acquired from Sigma-Aldrich (USA) and had a defined chemical identity and purity.

### 2.4. Super-Ovulation and Zygote Collection

Female mice were superovulated by intraperitoneal injection of 5 IU pregnant mare serum gonadotropin (PMSG; Sigma-Aldrich, St. Louis, MO, USA; G-4877), followed 48 h later by 5 IU human chorionic gonadotropin (hCG; Life Sciences, Seoul, Republic of Korea; 367222–1000 IU). The females were then paired with males of the same strain, and successful mating was confirmed the following morning by the presence of a vaginal plug. Approximately 16–18 h after hCG injection, females were euthanized by cervical dislocation. Oviducts were collected and transferred to HEPES-buffered F-10 medium (Gibco; Thermo Fisher Scientific, Waltham, MA, USA; 11550-043) supplemented with 10% serum synthetic substitute (SSS; Irvine Scientific, Santa Ana, CA, USA). Zygotes were released by tearing the ampulla, and cumulus cells were removed using hyaluronidase derived from bovine testes (Sigma-Aldrich, St. Louis, MO, USA; Cat. No. H3506). Cleaned embryos were washed and transferred to their respective culture media. To reduce any maternal effects, embryos obtained from each female were divided at random among all treatment groups.

### 2.5. Embryo Culture

F-10 medium supplemented with 10% serum synthetic substitute (SSS) and HEPES was used as the handling medium. The same F-10 medium was also used temporarily during embryo manipulation. For embryo culture, potassium simplex optimized medium (KSOM; Merck, Darmstadt, Germany; Cat. No. MR-121-D) served as the primary culture medium. KSOM droplets were used to culture embryos at 37 °C in a humidified incubator with 5% CO _2_ and atmosphere oxygen levels (approximately 20–21% O_2_).

### 2.6. Terminal Deoxynucleotidyl Transferase dUTP Nick End Labeling (TUNEL) Assay

Apoptotic cells in blastocysts were detected using the In Situ Cell Death Detection Kit (Roche Diagnostics, Indianapolis, IN, USA), following the manufacturer’s instructions [16]. Fixed embryos (n = 15 per group) were washed twice in 0.3% (*w*/*v*) polyvinylpyrrolidone (PVP) prepared in PBS (PVP–PBS) and permeabilized with 0.5% (*v*/*v*) Triton X-100 and 0.1% (*w*/*v*) sodium citrate for 30 min at room temperature. After permeabilization, embryos were washed and incubated with fluorescein-labeled dUTP and terminal deoxynucleotidyl transferase (TdT) at 37 °C for 1 h in the dark. Embryos were subsequently washed and counterstained with Hoechst 33342 (Hoechst 33342; Thermo Fisher Scientific, Waltham, MA, USA) (10 μg/mL) for 10 min. Following a final wash, blastocysts were mounted on glass slides for evaluation. Total cell numbers and apoptotic (TUNEL-positive) nuclei were counted under an epifluorescence microscope (Olympus IX71, Tokyo, Japan). TUNEL-positive nuclei appeared as bright red fluorescence, indicating apoptotic cells.

### 2.7. Real-Time PCR Analysis of Target Genes

The expression levels of GLUT4 and PI3K were analyzed by quantitative real-time PCR (qPCR). qPCR reactions were performed in duplicate using a CFX96 instrument (Bio-Rad Laboratories, Hercules, CA, USA) in a 10 μL reaction mixture containing 0.2 μM of each primer (Table 1), 1× iQ SYBR Green Supermix (Bio-Rad Laboratories), and 3 μL of diluted cDNA. Glyceraldehyde-3-phosphate dehydrogenase (GAPDH) was used as the internal reference gene. After confirming that GAPDH expression did not vary significantly among samples, relative transcript levels were quantified using independent qPCR reactions. Thermal cycling conditions were as follows: initial denaturation at 95 °C for 3 min; 44 cycles of 95 °C for 15 s, 57 °C for 20 s, and 72 °C for 30 s; followed by a final extension at 72 °C for 5 min. A melting curve analysis was performed from 65 °C to 95 °C at a rate of 0.2 °C/s, with fluorescence recorded continuously during the temperature increase. Relative gene expression was calculated using the ΔΔCt method after normalization to GAPDH expression. Intra- and inter-assay coefficients of variation (CVs) were determined using the formula: (standard deviation/mean) × 100 for all genes analyzed.

### 2.8. Statistical Analysis

Data, including total cell numbers per blastocyst, were analyzed using Prism 5 software (GraphPad Software Inc., San Diego, CA, USA). Differences between groups were evaluated using an unpaired two-tailed *t*-test or one-way analysis of variance (ANOVA), as appropriate. A value of *p* < 0.05 was considered statistically significant. Data are presented as the mean ± standard error of the mean (SEM), whereas cleavage and blastocyst development rates are expressed as percentages.

## 3. Results

### 3.1. Evaluation of Survival and Hatching Rates of Blastocysts

**Figure 1:**
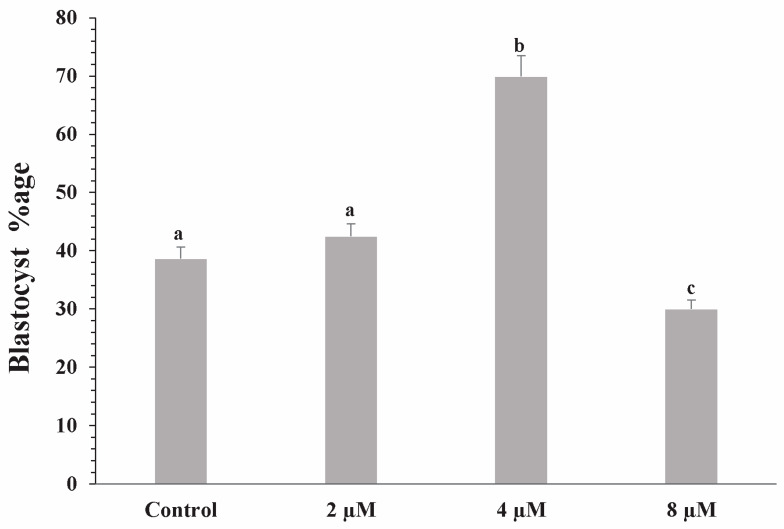
Developmental rates (%) of mouse blastocysts treated with different concentrations of anthocyanin. The bar graph shows the percentage of embryos that developed to the blastocyst stage in control and anthocyanin-treated groups. Data are presented as mean ± SEM. Different superscript letters (a–c) above the bars indicate statistically significant differences between groups (*p* < 0.05).

**Figure 2:**
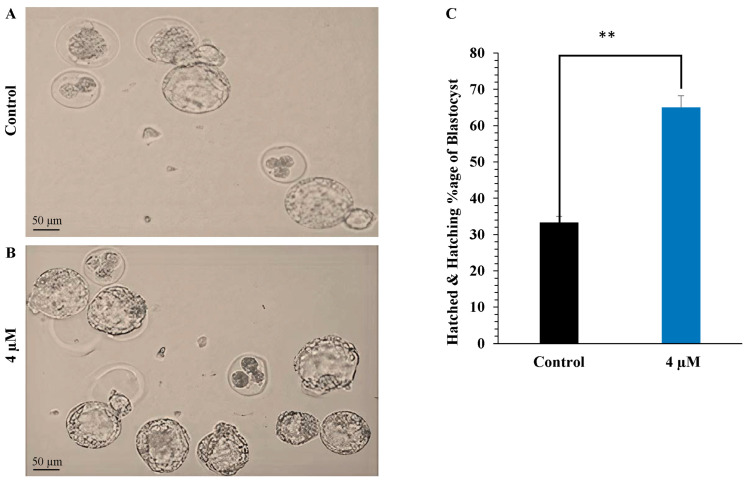
(**A**) Representative light microscope images of mouse blastocysts from the control group. (**B**) Representative images of blastocysts treated with 4 µM anthocyanin. (**C**) Percentage of hatched and hatching blastocysts in each group. Treatment with 4 µM anthocyanin significantly increased the hatching rate compared with the control group (** *p* < 0.01). Data are presented as mean ± SEM.

To evaluate blastocyst survival and hatching rates, embryos collected from ICR mice were cultured in KSOM medium for five days either without anthocyanin (control group) or with 4 μM anthocyanin, selected as the working concentration after preliminary optimization. After five days of culture, the anthocyanin-treated group exhibited higher survival and hatching rates compared with the control group (Figure 1 and Figure 2). A total of 600 embryos were collected from 120 mice; 400 embryos were used in preliminary concentration-optimization experiments, while the remaining 200 embryos were allocated to the control and 4 μM anthocyanin-treated groups for the main comparative analyses.

### 3.2. Counting of Total Cell Numbers and Apoptotic Cells

**Figure 3:**
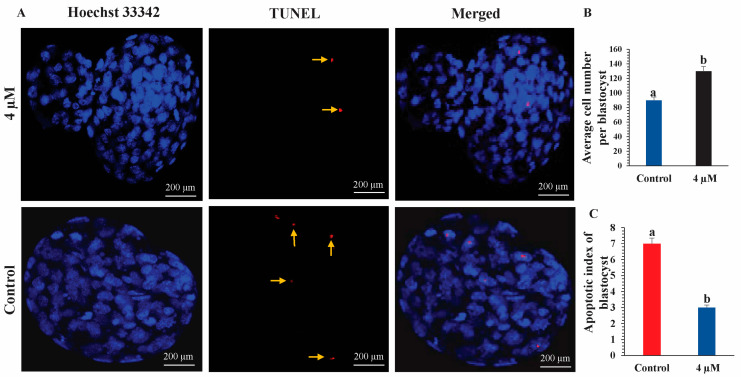
(**A**) Representative images of blastocysts from the control and 4 µM anthocyanin-treated groups are shown on the left. Nuclei are stained with Hoechst 33342 (blue), and apoptotic cells appear as red fluorescence, with arrows indicating TUNEL-positive nuclei. (**B**) Quantitative analysis of the average total cell number per blastocyst, which was significantly higher in the anthocyanin-treated group than in the control group (*p* < 0.05). (**C**) Apoptotic index of blastocysts, which was significantly lower in the anthocyanin-treated group compared with the control (*p* < 0.05). Data are presented as mean ± SEM, and different letters denote statistically significant differences.

Blastocyst quality was assessed by measuring the total cell number and the number of apoptotic cells in both the control and anthocyanin-treated groups. Treatment with 4 μM anthocyanin increased the total cell number and reduced the apoptotic index compared with the control group, indicating improved blastocyst quality (Figure 3).

### 3.3. mRNA Expression of Candidate Genes

**Figure 4:**
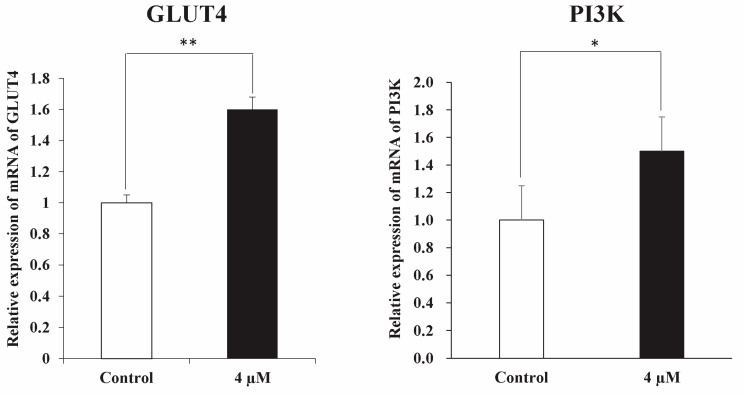
The bar graph on the left shows that treatment with 4 µM anthocyanin significantly increased the relative mRNA expression of GLUT4 compared with the control group (*p* < 0.01). The bar graph on the right shows that PI3K mRNA expression was also significantly higher in the anthocyanin-treated group than in the control group (*p* < 0.05). Data are presented as mean ± SEM. Statistical significance is indicated as * *p* < 0.05 and ** *p* < 0.01.

Quantitative PCR was performed to assess the expression of the candidate genes PI3K and GLUT4 in anthocyanin-treated and untreated samples. Gene-specific primers were used, and expression levels were normalized to the housekeeping gene GAPDH. The mRNA expression levels of both PI3K and GLUT4 were significantly higher in the anthocyanin-treated group compared with the untreated control group (*p* < 0.05) (Figure 4).

### 3.4. Proposed Mechanism of Anthocyanin-Mediated Blastocyst Development

**Figure 5:**
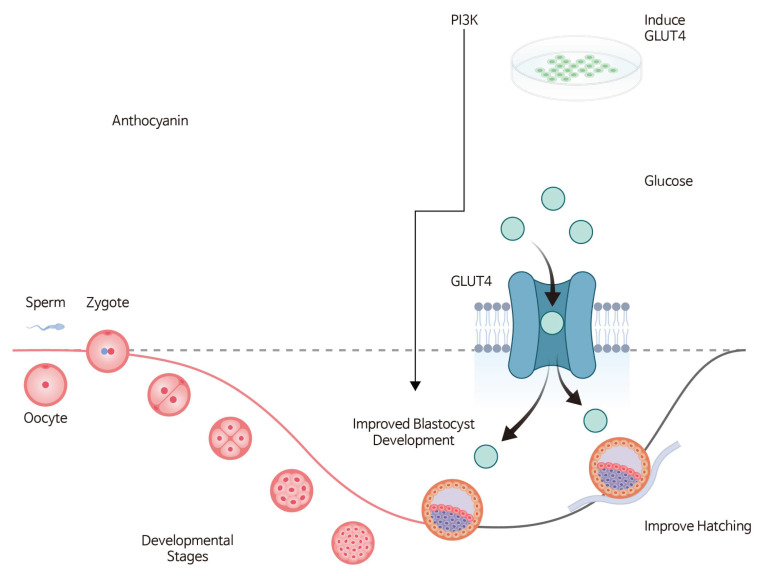
Schematic illustration of the proposed and hypothetical mechanism by which anthocyanin enhances blastocyst development and hatching. Anthocyanin supplementation is associated with increased mRNA expression of PI3K and GLUT4, which may be linked to enhanced glucose uptake and metabolic readiness. Improved glucose-related metabolic processes are proposed to support blastocyst development and facilitate hatching under in vitro conditions. All arrows and labels in the schematic represent proposed associations rather than experimentally validated causal relationships.

A schematic representation summarizing the proposed mechanism by which anthocyanin enhances blastocyst development and hatching is shown in Figure 5.

## 4. Discussion

Anthocyanins are violet plant pigments found in a variety of fruits, vegetables, and medicinal plants. They exhibit diverse physiological activities, including antioxidant, antidiabetic, and anticancer effects. Previous studies have shown that anthocyanins can modulate key genes involved in cellular metabolism, proliferation, and survival, such as phosphoinositide 3-kinase (PI3K) and glucose transporter type 4 (GLUT4) [17,18]. These properties make anthocyanins particularly relevant for embryonic development, where metabolic efficiency and cellular viability are critical.

Blastocyst quality is a major determinant of implantation success and subsequent pregnancy. In bovine species, high-quality (Grade I) blastocysts show markedly higher pregnancy rates (53%) compared with lower-quality blastocysts (36%) [19], highlighting the importance of embryo quality for reproductive outcomes. In the present study, anthocyanins were added to KSOM medium for the first time in a mouse embryo model. Supplementation with 4 µM anthocyanins significantly improved blastocyst quality, as demonstrated by an increased total cell number and a reduced apoptotic index relative to untreated controls. These findings suggest that anthocyanins enhance blastocyst viability by promoting cell survival and suppressing apoptosis, both of which are essential for normal preimplantation development in vitro. It needs to be mentioned that the first category of embryos, which we selected, was morphologically normal, and these embryos were randomly divided into two groups, one treated with anthocyanin and the other left untreated as the control group. Therefore, the results of our research primarily reflect the effects of anthocyanin on healthy embryos under optimized in vitro conditions. Although these findings suggest the potential of anthocyanins to improve blastocyst quality, future research should extend this work to evaluate their effectiveness in low-quality embryos and under conditions that more closely resemble clinical assisted reproductive technology (ART) settings, where embryo viability is of utmost concern.

Blastocyst hatching is a prerequisite for successful implantation, as the embryo must escape from the zona pellucida before attaching to the uterine epithelium. The timing of hatching is crucial; early hatching enhances uterine receptivity and increases the likelihood of implantation, while delayed hatching may reduce the implantation window [20]. Our findings demonstrated that 4 µM anthocyanin supplementation improved the hatching rate of mouse blastocysts in vitro. This improvement may result from enhanced enzymatic activity involved in zona pellucida digestion or from improved mechanical properties of the blastocyst, facilitating more efficient hatching. Thus, anthocyanins may support implantation by improving both embryo survival and hatching competence.

The PI3K/Akt signaling pathway plays a central role in regulating cell proliferation, survival, and metabolic activity. Activation of PI3K induces phosphorylation of Akt, which subsequently activates cyclin-dependent kinases (CDKs) required for normal cell-cycle progression [21]. This pathway is vital for maintaining cellular functions during embryonic development. In bovine embryos exposed to heat stress, activation of PI3K signaling has been shown to enhance blastocyst development and reduce apoptosis [16]. Consistent with these findings, our study showed that 4 µM anthocyanin supplementation upregulated PI3K expression in mouse blastocysts, supporting improved cell survival and developmental competence. These results suggest that anthocyanins may enhance embryo development by modulating PI3K-mediated pathways.

GLUTs are membrane proteins that mediate glucose uptake, and GLUT4 is one of the most extensively studied members of this family. It plays a crucial role in glucose transport in insulin-responsive tissues such as adipose tissue and the uterus [22]. Dysregulation of GLUT4 expression has been associated with metabolic disorders including type 2 diabetes and polycystic ovary syndrome (PCOS), both of which are linked to increased miscarriage risk due to impaired glucose metabolism [23,24]. GLUT4 is also essential for implantation, as it is strongly expressed in the endometrium of pregnant mice from Day 1 to Day 4 post-fertilization [25]. In this study, anthocyanin treatment significantly increased GLUT4 expression in blastocysts relative to controls. This upregulation suggests that anthocyanins may improve glucose uptake and metabolic activity in preimplantation embryos, providing the energy required for continued growth. Elevated GLUT4 expression in anthocyanin-treated blastocysts therefore indicates enhanced metabolic readiness, which may support preimplantation embryo development.

These findings suggest that anthocyanins may influence preimplantation embryo development, potentially involving PI3K-mediated signaling and glucose-related pathways. Although the present study assessed changes at the mRNA expression level, the observed upregulation of PI3K and GLUT4 transcripts indicates a possible association with enhanced cell survival and metabolic readiness. However, it should be noted that mRNA expression does not necessarily translate into functional protein activity, and these effects require validation at the protein level and through functional or in vivo assays in future studies. Overall, this study provides preliminary insight into the potential biological effects of anthocyanins during early embryo development and may serve as a basis for further research aimed at optimizing embryo culture conditions in reproductive technologies.

## 5. Conclusions

This study demonstrates that anthocyanin supplementation improves the developmental competence and hatching rate of ICR mouse blastocysts under in vitro conditions. Anthocyanin treatment also upregulated the expression of GLUT4 and PI3K, at the mRNA level, suggesting a potential association with enhanced glucose metabolism and cellular proliferation. These results indicate that anthocyanins may serve as a beneficial natural compound for improving embryo quality during preimplantation development in vitro. Further studies are required to elucidate the underlying molecular mechanisms and to validate these findings at the protein and functional levels, as well as to assess their relevance in in vivo or clinical reproductive settings.

## Figures and Tables

**Table 1:** Primer sequences used to analyze expression of target and housekeeping genes.

Genes	Primer Sequence
*PI3K*	F: 5′-CTCTCCTGTGCTGGCTACTGT-3′ R: 5′-GCTCTCGGTTGATTCCAAACT-3′
*GLUT4*	F: 5′-GTAACTTCATTGTCGGCATGG-3′ R: 5′- AGCTGAGATCTGGTCAAACG-3′
*GAPDH*	F: 5′-TGTGTCCGTCGTGGATCTGA-3′ R: 5′-TTGAGGGTGCAGTGATGGG-3′

Abbreviations: F, forward; R, reverse.

## Data Availability

The data presented in this study are available from the corresponding author upon reasonable request.
